# A preliminary investigation of circulating extracellular vesicles and biomarker discovery associated with treatment response in head and neck squamous cell carcinoma

**DOI:** 10.1186/s12885-019-5565-9

**Published:** 2019-04-23

**Authors:** Dorival Mendes Rodrigues-Junior, Soon Sim Tan, Luciano de Souza Viana, Andre Lopes Carvalho, Sai Kiang Lim, N Gopalakrishna Iyer, Andre Luiz Vettore

**Affiliations:** 1 Department of Biological Science, Laboratório de Biologia Molecular do Câncer, UNIFESP, Universidade Federal de São Paulo, Rua Pedro de Toledo, 669 – 11° andar, São Paulo, SP 04039-032 Brazil; 2 Cancer Therapeutics Research Laboratory, National Cancer Centre of Singapore, 11 Hospital Drive, Singapore, 169610 Singapore; 3 Institute of Medical Biology, A*-STAR, Singapore, Singapore; 4 Molecular Oncology Research Center, Barretos Cancer Hospital, Barretos, SP Brazil; 5 Division of Surgical Oncology, National Cancer Centre of Singapore, Singapore, Singapore

**Keywords:** Biomarker discovery, Chemoradiation therapy, Extracellular vesicles, Head and neck squamous cell carcinoma, HNSCC, Treatment response

## Abstract

**Background:**

There is a paucity of plasma-based biomarkers that prospectively segregate the outcome of patients with head and neck squamous-cell carcinoma (HNSCC) treated with chemoradiation therapy (CRT). Plasma extracellular vesicles (EVs) might be an alternative source for discovery of new specific markers present in patients with HNSCC, which could help to re-direct patients to appropriate curative therapies without delay.

**Methods:**

In order to identify new markers in plasma compartments, *Cholerae* toxin B chain (CTB) and Annexin V (AV) were used to isolate EVs from pooled plasma samples from patients with locally advanced HNSCC who responded (CR, *n* = 6) or presented incomplete response (NR, n = 6) to CRT. The crude plasma and EVs cargo were screened by antibody array.

**Results:**

Of the 370 polypeptides detected, 119 proteins were specific to NR patients while 38 were exclusive of the CR subjects. The Gene Set Enrichment Analysis (GSEA) and Search Tool for the Retrieval of Interacting Genes (STRING) database analysis indicated that the content of circulating plasma EVs might have a relevant function for the tumor intercellular communication in the HNSCC patients.

**Conclusion:**

This study provides a list of potential markers present in plasma compartments that might contribute to the development of tools for prediction and assessment of CRT response and potentially guide therapeutic decisions in this context.

**Electronic supplementary material:**

The online version of this article (10.1186/s12885-019-5565-9) contains supplementary material, which is available to authorized users.

## Background

Head and neck cancer affects 740,000 new patients each year, with approximately 300,000 deaths worldwide [1, 2], of which more than 90% are histologically squamous (HNSCC). Despite significant advances in therapy, only 30–50% of patients with locally advanced disease survive more than 5 years, and this has not changed over the past 40 years [3]. In this context, concurrent chemoradiation therapy (CRT), with or without induction chemotherapy (IC), has emerged as the new paradigm of treatment for patients with locally advanced HNSCC affecting the oropharynx, larynx and hypopharynx [4]. While the objective of this strategy is to preserve critical functions (airway, speech, swallowing, body image, etc.), a proportion of patients fails treatment and requires salvage surgery. Noteworthy, ineffective pre-treatment results in unwanted delay of curative therapy and in addition, surgery is made challenging in a heavily pre-treated patient and in a radiated field [5]. It is generally believed that personalized treatment decisions based in non-invasive biomarkers able to stratify patients according to treatment response would help to optimize appropriate patient-specific therapeutic interventions, quality of life and outcomes.

The discovery of extracellular vesicles (EVs) could circumvent these issues and allow analyses of a much ‘cleaner’ plasma compartment that may better reflect the tumor cells or its microenvironment. EVs are nanosize bi-lipid membrane vesicles carrying various cellular components (proteins, lipids, nucleic acids, and sugars) and released by cells into the extracellular space [6, 7]. The EVs obtained from plasma of cancer patients are enriched in proteins/glycoproteins expressed on cell membranes and/or in the cytosol of the parent tumor cells, and in many immunosuppressive molecules [8]. The EVs are involved in the transmission of biological signals between different populations of cells [9].

There are emerging evidences that cancer-derived EVs play a crucial role in the reprogramming of both local microenvironment and distant sites, contributing to tumor capacity for immune evasion, growth, invasion and metastatic spread [10, 11]. Furthermore, since they are secreted into the intercellular space, EVs can be detected in a variety of biological fluids, including saliva, cerebrospinal fluid, urine and serum/plasma. The potential application of EVs for diagnosis and guiding therapeutics, as well as determining prognosis of pathological conditions has allowed the field of EV-studies to grow steadily in recent years [12]. Of note, HNSCC are strong EVs producers and the plasma of patients with HNSCC is highly enriched in EVs [13, 14].

Based on the potential role of tumor-derived EVs as mediators of tumorigenesis, others and we have reasoned that the molecular content of plasma EVs makes them potential biomarker sources [15–17]. Therefore, here we propose the use of antibody arrays to identify and quantify proteins carried by EVs circulating in the plasma of HNSCC patients that do not respond to CRT (NR, Non-Responders), and compared this profile with patients that present a complete response to this treatment (CR, Complete-Responders).

So, in this study, we identified several plasma biomarker candidates present in plasma compartments with the potential to predict beforehand which patients could take benefit from CRT and to guide therapeutic decisions in this context.

## Methods

### Patients

This study involved plasma samples from 12 patients with locally advanced HNSCC who underwent organ preservation protocol as part of a phase 2 clinical trial conducted to test the effect of IC followed by CRT, between 2009 and 2010 at the Department of Head and Neck Surgery, Barretos Cancer Hospital (Barretos, SP, Brazil) [4]. This study was approved by the institution ethics committees (CEP-UNIFESP: 1610/2016; CEP-HCBarretos: 231/2009). Inclusion criteria were: histologically confirmed locally advanced stage III or IV a-b (M0) squamous cell carcinoma of the larynx, oral cavity, or oropharynx with no prior treatment or cancers, and informed consent to undergo the treatment as outlined. All patients were required to have measurable disease by Response Evaluation Criteria in Solid Tumors (RECIST, version 1.1), an Eastern Cooperative Oncology Group-Performance Status ≤2, age ≥ 18 years, and adequate liver, renal, and bone marrow function. Patients with oral cavity cancer were included only if they had unresectable disease. Exclusion criteria included a history of another malignancy, previously received chemotherapy, radiotherapy, or surgery (except diagnostic biopsy) for the primary tumor or lymph nodes, presence of a serious concomitant illness and a psychiatric illness that would preclude the delivery of the treatment.

A previous definition of tumor response to treatment and disease progression was used in this study [4, 18]. Briefly, tumor response was determined by clinical evaluation, imaging studies (performed at baseline, 2 weeks after the third cycle of induction chemotherapy, and 6–8 weeks after the end of radiotherapy) and biopsy/surgery (when indicated). An independent review of radiologic data was performed. Tumor response to treatment was considered as complete response (CR) when there was disappearance of all detectable lesions, and non-response (NR) as tumor response less than complete (partial response or stable disease), or progression of disease - the appearance of a new lesion, or increase of any lesion classified as measurable at initial examination (including tumor recurrence), the definition followed the RECIST 1.1 criteria. Patients with documented persistent/residual disease after completing CRT were also eligible to undergo salvage surgery (for the primary tumor or the neck) or palliative care. More details of the trial, including the definition of tumor response can be found in the previously published results [4].

### EVs isolation

It was described that EVs are enriched in specific phospholipids such as GM1 ganglioside and phosphatidylserine, which has high specific binding to the *Cholerae* Toxin chain B (CTB) and to Annexin V (AV), respectively [19, 20]. Subpopulations of EVs were isolated from the plasma of CR or NR patients using beads coated with CTB and AV as previously described by [20]. Briefly, due to the scarcity of plasma from HNSCC patients, 50 μL from each plasma sample were pooled to have 2 distinct pools, one for the CR cases and other for the NR ones (6 samples were added to each pool). One hundred microliters of each pool were incubated with 0.5 μg of biotinylated CTB (#C34779; ThermoFisher) or with 0.5 μg of biotinylated AV (#K109, Biovision) dissolved in 100 μl of PBS or AV binding buffer for 60 min at 37 °C. At the same time, 50 μL of Dynabeads MyOne Streptavidin T1 (#65602; ThermoFisher) were washed three times with 100 μL wash buffer (0.1% bovine albumin in PBS). Finally, the beads were resuspended in 100 μL of the PBS filtered in a 0.22 μm filter. Fifty microliters of beads (CTB or AV) were added to the plasma mixture and incubated for 30 min at 25 °C. The magnetic beads were immobilized with a magnet, washed three times with 200 μL of PBS and the isolated EVs bound to CTB or AV were stored at − 20 °C.

### Antibody Array

For antibody array, CTB- and AV-EVs isolated from CR and NR plasma pools were lysed with cell lysis buffer (#K269; Biovision) and 100 μL of the protein lysate were analyzed using the *Fullmoon Biosystems Explorador Antibody* (#ASB600, Fullmoon Biosystems) according to manufacturer’s instructions. We also conducted the same analysis with 100 μL of crude plasma (without EVs isolation) from both patient pools. After the immune reaction, following the manufacturer’s recommendations, the arrays were scanned and the values were normalized using GenePix Pro 7 software (Molecular Devices) to correct for any technical, chip-to-chip, or day-to-day variations. Since in the matrix there were two replicates of each spot, the relative expression means between the replicates were calculated. The reactivity against the controls contained in each matrix was used as background cutoff, and the reactivity higher than the background was classified as present and the lower reactivity as absent.

### Statistical analyses

The chi square exact test was used to evaluate the associations between chemoradiation therapy response and clinical variables. The specific proteins present in the EV isolated from plasma of HNSCC CR and NR patients (CTB- and AV-EVs) were functionally clustered using the PANTHER (Protein ANalysis THrough Evolutionary Relationships, http://pantherdb.org) algorithm by estimating the hypergeometric distribution of overlapping genes and, based on their connectivity, biological networks were algorithmically generated using this software [21]. The *P* values were calculated with Fisher’s exact test with FDR multiple test correction and only proteins having q < 0.05 were used in the analysis. To identify those proteins that were associated with tumorigenesis processes, the Gene Set Enrichment Analysis (GSEA) algorithm (http://software.broadinstitute.org/gsea/msigdb) was used. Moreover, a protein-protein interaction network for the specific proteins present in EVs according to treatment response was constructed using the database: Search Tool for the Retrieval of Interacting Genes (STRING - version 10.5; http://string-db.org/), with the required high confidence score (> 0.7). Subsequent KEGG pathway enrichment analyses were performed.

## Results

### Patient characteristics

The clinical and histological features of the 12 patients with locally advanced HNSCC enrolled in this study are presented in Table 1. The median follow-up for this cohort was 4 years. The patients were only males, with age ranging from 37 to 68 years (median: 51.5 years). Tobacco use (current and former) was reported by 91.7% (*n* = 11) of the patients, while only 8.3% of the cases were HPV-associated cancers (n = 1). Primary tumor sites included oral cavity (*n* = 2; 16.7%), oropharynx (*n* = 8; 66.7%), and larynx (n = 2; 16.7%) and 75% of the HNSCC were classified as T stage IV. Intentionally, six patients (50.0%) presented complete response to CRT while six ones (50.0%) did not respond to the treatment. None of the clinical variables were correlated with the chemoradioresistant tumors.

**Table 1:** Clinical and pathologic characteristics of the patients included in the study. All *p* values were based on 2-tailed chi square test

Variables		N of cases (%)	CR n (%)	NR n (%)	X^2^ (*p* value)
Gender	Male	12 (100.0)	6 (50.0)	6 (50.0)	1.0
	Female	0 (0)	0 (0)	0 (0)	
Age	<=60 years	10 (83.3)	5 (41.7)	5 (41.7)	1.0
	> 60 years	2 (16.7)	1 (8.3)	1 (8.3)	
Tobacco Consumption	yes	11 (91.7)	6 (50.0)	5 (41.7)	0.2963
	no	1 (8.3)	0 (0)	1 (8.3)	
Tumor site	Oral Cavity	2 (16.7)	0 (0.0)	2 (16.7)	0.6283
	Oropharynx	8 (66.7)	4 (33.3)	4 (33.3)	
	Larynx	2 (16.7)	2 (16.7)	0 (0.0)	
HPV status^a^	p16+	1 (8.33)	1 (8.3)	0 (0.0)	0.2963
	p16-	11 (81.7)	5 (41.7)	6 (50.0)	
Tumor stage	III	3 (25.0)	3 (25.0)	0 (0)	0.1824
	IV	9 (75.0)	3 (25.0)	6 (50.0)	

Abbreviations: *CR* complete response to chemoradiotherapy, *NR* incomplete response to chemoradiotherapy

^a^HPV status was determined retrospectively using archival tumor specimens

### Identification of differences in the protein cargo of EVs circulating in the plasma of CR and NR patients

Blood plasma samples collected from two types of subjects, chemoradioresistant (NR) and chemoradiosensitive (CR) patients, were pooled into two distinct pools according to the treatment response and incubated with either biotinylated CTB or AV molecules. Isolated CTB-EVs and AV-EVs were lysed and the total protein content was tested against a commercial explorer antibody array for biomarker candidates.

**Fig. 1:**
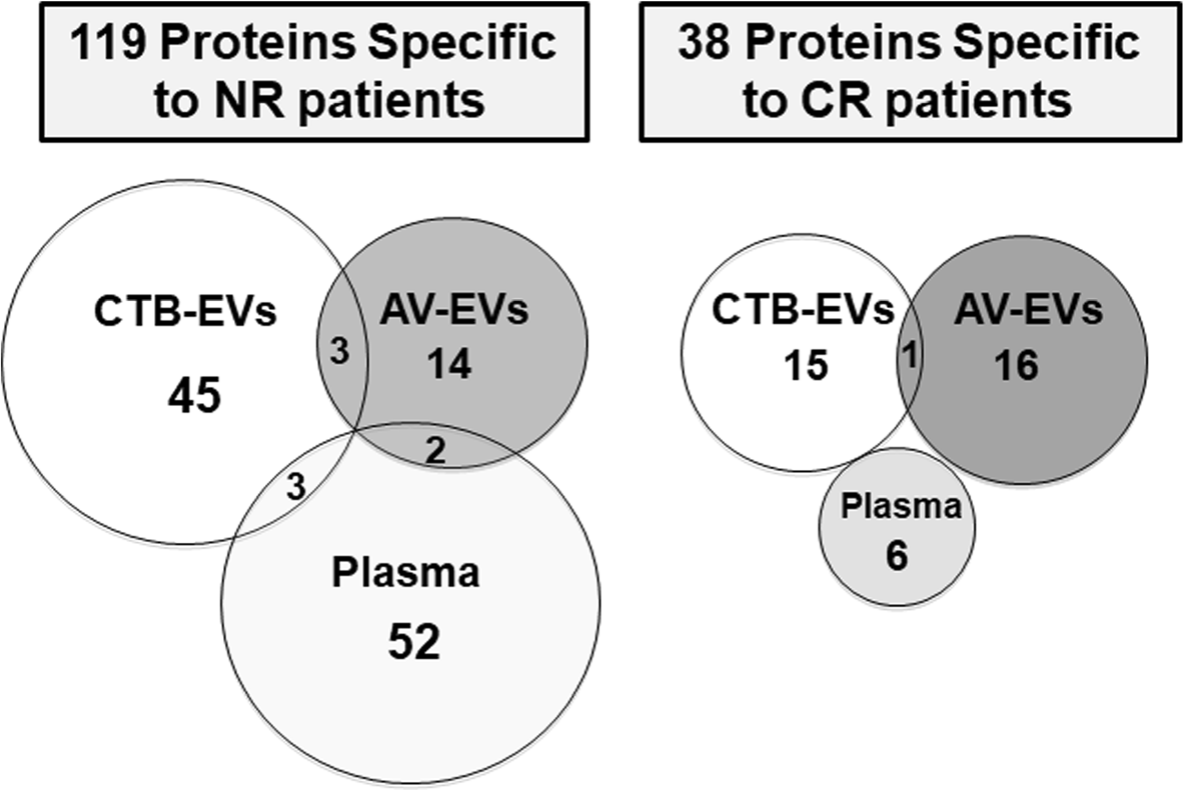
Distribution of specific proteins in the CTB-EVs, AV-EVs and crude plasma detected through Antibody Array assays. The comparison of the protein content in CTB-EVs, AV-EVs and crude plasma from pooled 6 complete responder (CR) patients and 6 non-responder (NR) patients revealed 119 proteins specific to the NR patients, while 38 proteins were specific to the CR patients. The level of protein detected was normalized by the mean of positive controls present in the antibody array assay (Full Moon Biosystems)

From the 656 antibodies immobilized in the array, we were able to detect the presence of a total of 370 proteins present in the plasma samples from the HNSCC patients. From that, 119 proteins were specific to NR patients while 38 were exclusive to CR subjects (Fig. 1). Of the 119 proteins detected in the plasma samples of the non-responder patients (chemoradioresistant), 45 were found exclusively in the CTB-EVs, 14 were observed only in the AV-EVs, while 52 were not carried by either EV types. Three proteins were presented in both subpopulations of EVs (CD4, POLB and PRIM1), three proteins were detected in CTB-EVs and crude plasma (IFNG, MMP1 and VHL), while two ones were present in AV-EVs and crude plasma (HLA-DP and HSP90AB1) (Additional file 1: Table S1). Of the proteins detected in the plasma of the patients who responded to the CRT (chemoradiosensitive), 15 were found only in the CTB-EVs, 16 were identified in the AV-VEs solely, SNAI1 was detected in both CTB- and AV-EVs, and 6 proteins could only be detected in the crude plasma samples (Additional file 2: Table S2). Forty-three proteins of the 119 proteins (36.13%) detected in plasma of NR patients and 14 of 38 (36.84%) present in the CR cases are associated with tumorigenesis processes according to GSEA database (Additional file 1: Table S1; Additional file 2: Table S2). According to this analysis, EVs derived from NR patients carry previously reported tumor biomarker candidates such as: FAS, RET, STAT5, TNFRSF1B, WNT1, ABCB1, CASP5, CCND1, FGF1, ABL1, BCL2L1, PRIM1, CD4, HSP90AA1 and HSP90AB1, while BAX, CASP3, HDAC1, NGFR, TNFSF11, TP73, BRCA2, EGFR, IKBKB, STAT1, SNAI1, BAG1 and TNFRSF10B were detected in EVs from CR patients.

**Fig. 2:**
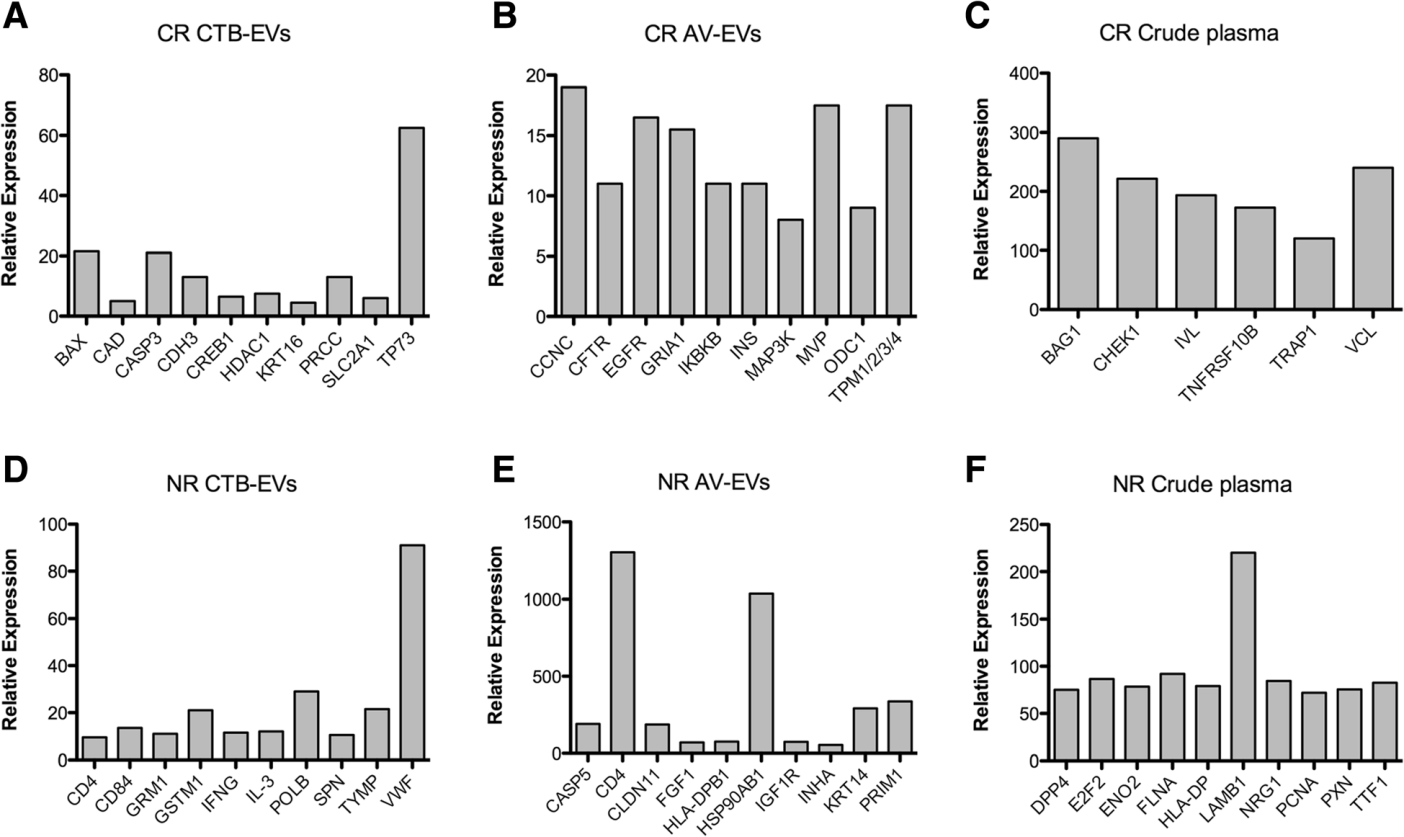
Relative abundances of the putative HNSCC protein biomarkers for CRT treatment response in the pooled plasma samples analyzed**.** Among the specific proteins detected in each subpopulation (CTB-EVs, AV-EVs and crude plasma), the top 10 most abundant ones in patients with complete response to CRT (CR; **a**, **b** and **c**) or from the non-responders (Nr; **d**, **e** and **f**) are presented

The distributions of the protein abundance in the CTB-EVs, AV-EVs and crude plasma are presented in Fig. 2. The most abundant proteins in CR patients were BAX, CASP3, TP73 (in CTB-EVs), CCNC, MVP, ODC1 (in AV-EVs), BAG1, CHEK1, and VCL (in the crude plasma). Likewise, the plasma of the NR patients was rich in POLB, TYMP, VWF (in CTB-EVs), CD4, HSP90AB1 (in AV-EVs), and LAMB1 (in the crude plasma).

### Canonical pathway analysis and protein-protein interaction networks

The proteins identified in the CTB- and AV-EV cargos of the CR and NR, groups were functionally clustered into key pathways using the PANTHER algorithm to determine if they contributed to the respective response to CRT. The EVs proteins from the CR patients (*n* = 32) and the NR ones (*n* = 67) were found clustered into 16 statistically significant pathways (Table 2). In the CR patients, the most prominently cancer networks were related to FAS, p53, apoptosis, and Cadherin signaling pathways. On the other hand, of the nine pathways in which the circulating EV proteins from NR patients clustered, six were tumorigenesis-signaling pathways (MAP kinase, PI3K/AKT, Interleukin VEGF, EGF, and angiogenesis).

**Table 2:** List of significant pathways identified through PANTHER database (Protein ANalysis THrough Evolutionary Relationships) with the protein carried by extracellular vesicles circulating in the plasma of HNSCC patients, according to CRT response (*CR* complete responders or *NR* non-responders)

Related Pathway	Raw *p*-value	FDR q-value	Related Molecules
NR EVs (n of proteins = 67)			
Insulin/ MAP kinase cascade	1.85E-04	7.54E-03	IGFR1, RAF1, IRS1
PI3 kinase	7.62E-04	1.38E-02	NOS3, CCND1, IRS1
Interleukin signaling	1.95E-04	6.35E-03	NOS3, RAF1, IRS1, STAT5
VEGF signaling	1.37E-03	2.24E-02	VEGFA, NOS3, RAF1
EGF receptor signaling	8.47E-05	6.90E-03	YWHAB, RAF1, ERBB4, GAB1, STAT5
Alzheimer disease-presenilin	6.37E-04	1.30E-02	MMP1, CTNNA1, ERBB4, WNT1
Angiogenesis	2.21E-04	6.01E-03	MMP1, CTNNA1, ERBB4, WNT1
CCKR signaling map	2.27E-04	5.29E-03	ITGB1, YWHAB, RAF1, IRS1, CCND1
Gonadotropin-releasing	1.02E-04 5.57E-03		ITGB1, MT-CO2, INHA, RAF1, IGFR1, IRS1
CR EVs (n of proteins = 32)			
FAS signaling p53 signaling	1.63E-03	4.43E-02	CAD, CASP3
	4.76E-04	1.94E-02	TP73, BAX, HDAC1
Apoptosis signaling	5.19E-05	2.82E-03	BAX, IKBKB, CASP3, CREB1
CCKR signaling map	1.10E-05	8.99E-04	ODC1, SNAI1, BAX, CREB1, CASP3
Huntington disease	1.88E-03	4.39E-02	TP73, BAX, CASP3
Cadherin signaling	2.39E-03	4.88E-02	CDH3, EGFR
Gonadotropin-releasing	6.61E-04	2.15E-02	CREB1, EGFR, INS, SLC2A1

A protein-protein interaction network was acquired using the STRING database. The network of specific proteins of CTB- and AV-EVs from CR patients (n = 32) comprised 19 nodes and 26 edges. The top five KEGG pathways observed were pathways in cancer, hepatitis B, pancreatic cancer, prolactin signaling pathway and prostate cancer (Additional file 3: Figure S1). The network for specific proteins of CTB- and AV-EVs from NR patients (n = 67) showed 43 nodes and 72 edges (Additional file 4: Figure S2.). The top five KEGG pathways observed for these proteins were pathways in cancer, PI3K/AKT signaling pathway, proteoglycans in cancer, antigen processing and presentation and bladder cancer.

## Discussion

Over the last years, management and prognosis of patients with HNSCC has improved through multimodality treatment protocols, which includes surgery, radiotherapy and chemotherapy, but long-term survival rate is still around 50% [3]. Thus, to improve the prognostic of HNSCC patients by selecting the most appropriate curative therapies, this study aimed to identify novel non-invasive candidate biomarkers in plasma EVs that could segregate locally advanced HNSCC patients who respond to chemoradiation therapy from those who do not*.*

Different approaches have been used to identify prognostic factors in HNSCC. Many are based on analyses of the primary tumor tissue itself. However, a few studies have focused on plasma for discovery of prognostic biomarkers in HNSCC. Le et al. (2003) [22] reported that serum Osteopontin levels correlated with tumor hypoxia, and suggested that this protein could be used to identify patients at high risk for tumor recurrence. Similarly, Gehrmann et al. (2014) [23] showed that serum Hsp70 levels in the HNSCC patients might be useful for tumor detection, and also for monitoring therapeutic response to radiation therapy. In other studies, plasma levels of LCN2/matrix metallopeptidase 9 complex, MMP2, TIMP1, TIMP2 and TIMP3 were correlated to tumor size, lymph node involvement, tumor differentiation and prediction of tumor stage and T status in patients affected with HNSCC [24–26]. However, none of these markers are being used as clinical predictors for outcome.

Antibody arrays are screening assays for the presence and absence of an antigen based on the highly specific recognition between an antibody and its target antigen. Several studies have reported the use of antibody arrays in cancer biomarker discovery studies to discover biomarkers that are potentially valuable for diagnosis, prognosis or treatment response evaluation. Sill et al. (2016) [27] used this approach to compare gastric cancer cells to adjacent normal tissue and they found 17 proteins with high specificity for gastric adenocarcinoma. Another antibody array study revealed that cyclin D2, cytokeratin 18, cyclin B1, hnRNP m3-m4 and the monophosphorylated ERK were decreased in a doxorubicin resistant breast cancer cell line in comparison to a sensitive one [28]. Sreekumar et al. (2001) [29] also used antibody array assays to identify up-regulated apoptotic factors in colon carcinoma treated with ionizing radiation.

Utilization of peripheral blood for the discovery of tumor biomarkers is a very practical approach because it is minimally invasive, inexpensive, highly reproducible, and it could mitigate the intra-temporal heterogeneity-sampling problem [30]. Zupancic et al. (2014) [31] analyzed non-depleted plasma samples of healthy volunteers in comparison to glioblastoma multiform (GBM) patients using antibody arrays and identified 11 plasma proteins as biomarker candidates for the diagnosis and prognosis of patients with GBM. Although plasma and serum are reliable sources for cancer biomarker discovery, its usage has several limitations as previously discussed [20]. One limitation is the presence of high-abundance proteins. Although plasma and serum have more than 10,000 proteins, > 99% of the serum protein mass is dominated by 21 proteins [32, 33]. Furthermore, depletion of these high abundance proteins to uncover low-abundance biomarkers might introduce artifacts and bias to proteins quantification [34]. The discovery of extracellular vesicles (EVs), carrying proteins and nucleic acids in various body fluids, such as plasma, is an alternative method to circumvent some of these limitations. In a previous study, we shown evidences that it is possible to isolate at least two distinct populations of EVs in plasma, according to their affinities for CTB or AV ligands, and these EVs might have different origins and molecular contents. Moreover, this method is highly specific for the isolation of phospholipid membrane vesicles with minimal contamination of large non-vesicular biological complexes or high abundant plasma proteins [20]. Importantly, these methods are able to capture EVs from relatively small plasma volumes and even from freeze-thawed plasma samples, with the potential for use either in large longitudinal studies or routine clinical use.

We therefore performed an antibody array analysis of CTB- and AV-EVs in a pool of plasma samples from HNSCC patients (responders and non-responders to CRT). This analysis revealed that EVs derived from NR patients carry previously reported tumor biomarker candidates. Patel et al. (2014) [35] showed that inhibition of HSP90 potentiated cell death in HNSCC cells induced by cisplatin and radiotherapy. The increased percentage of circulating CD4+ cells was found to predict response to induction chemotherapy in advanced laryngeal cancer [36]. Notwithstanding, it is well known that gene repair is one of the most relevant mechanisms of resistance to anti-cancer drugs and two proteins related to DNA repair, PRIM1 and POLB, were detected in both EV subpopulations (CTB and AV) in NR patients. Previously reported tumor biomarker candidates were also detected in the cargo of EVs circulating in the plasma of CR patients. Liu et al. (2014) [37] proposed that TP73, a protein involved in the signaling pathway of DNA damage, is a potential target for miRNAs to induce cisplatin resistance in ovarian cancer. It was also reported that BRCA2-negative ovarian cancer cells are more sensitive to cisplatin treatment [38]. The EGFR was found to be overexpressed in 90% of HNSCC patients and its high levels in the tumor tissue has been strongly correlated with worst clinical outcome [39]. In addition, the loss of STAT1 has been related to the tumorigenesis of several cancer types, and is implicated as a tumor suppressor in esophageal squamous cell carcinoma [40]. A significant correlation between the ratios of BAX/ BCL2 was reported to segregate radiosensitive versus radio-resistant breast cancer patients [41]. SNAI1, which is a master regulator of epithelial-to-mesenchymal transition, has shown to be overexpressed in several cancer cells and is often related to prognosis and aggressiveness [42]. The suppression of IKBKB through miR-16 sensitizes breast cancer cells to paclitaxel treatment [43].

In the present study, we identified a set of 119 proteins that were specific to the patients that do not respond to CRT. A fraction of proteins detected in the EVs from these patients were associated with Insulin/ MAP kinase and PI3 kinase signaling, and angiogenesis. It is noteworthy that angiogenesis plays a critical role in HNSCC progression and high plasma levels of the angiogenic factors, VEGF and EFGR, have been related to the worst prognosis of HNSCC patients [44, 45]. On the other hand, 38 different proteins were found specific to the HNSCC patients presenting a complete response to CRT. Among the most significant pathways associated with these proteins, were FAS, p53 and apoptosis cell death signaling, which are critical pathways in HNSCC patients associated with response to chemoradiation therapy [14, 46–48]. These pathway analyses suggest that the content of circulating plasma EVs could have relevant functions in the treatment response of HNSCC patients.

## Conclusions

In summary, we have identified biomarker candidates present in plasma EVs or crude plasma that could stratify HNSCC patients according to their response to CRT. However, these markers will have to be validated further in larger cohorts in larger scale studies. This study has a number of pitfalls, including a small patient cohort and availability of plasma samples from only a single time point. Nevertheless, this study provided proof of principle that screening plasma EV subpopulations using antibody arrays is a viable strategy for biomarker discovery.

## Additional files

Additional file 1: **Table S1** List of proteins present in CTB-, AV-EVs and crude plasma of non-responders HNSCC patients. Mean of relative expression was normalized using GenePix Pro 7 software (Molecular Devices). Gene Set Enrichment Analysis (GSEA) algorithm was performed to identify proteins positively related to cancer (+). (TIF 1022 kb)

Additional file 2: **Table S2** List of proteins present in CTB-, AV-EVs and crude plasma of complete responders HNSCC patients. Mean of relative expression was normalized using GenePix Pro 7 software (Molecular Devices). Gene Set Enrichment Analysis (GSEA) algorithm was performed to identify proteins positively related to cancer (+). (TIF 943 kb)

Additional file 3: **Figure S1** Protein-protein interaction network to specific proteins in EVs from CR patients. Predicted interactions for these proteins (*n* = 32) were obtained from STRING online database (http://string-db.org). The top five KEGG pathways observed in these proteins were pathways in cancer (red; eight proteins, *p* = 5.44 × 10^− 6^), hepatitis B (purple; five proteins, *p* = 0.0002), pancreatic cancer (green; four proteins, p = 0.0002), prolactin signaling pathway (yellow; four proteins, p = 0.0002) and prostate cancer (lilac; four proteins, *p* = 0.0004). (DOCX 39 kb)

Additional file 4: **Figure S2** Protein-protein interaction network construction by STRING to specific proteins in EVs from NR patients. Predicted interactions for these proteins (*n* = 67) were obtained from STRING online database (http://string-db.org). Top five KEGG pathways related to specific proteins in NR-EVs were pathways in cancer (red; 15 proteins, *p* = 1.74 × 10^− 11^), PI3K/AKT signaling pathway (purple; 12 proteins, *p* = 8.4 × 10^− 8^), proteoglycans in cancer (green; ten proteins, *p* = 1.58 × 10^− 7^), antigen processing and presentation (yellow; six proteins, *p* = 4.56 × 10^− 6^) and bladder cancer (lilac; five proteins, *p* = 5.34 × 10^− 6^). (DOCX 25 kb)

## Abbreviations

AV: Annexin V
CR: Complete-Responders
CRT: chemoradiation therapy
CTB: Cholerae toxin B chain
EVs: Extracellular vesicles
GSEA: Gene Set Enrichment Analysis
HNSCC: Head and neck squamous-cell carcinoma
IC: Induction Chemotherapy
NR: Non-Responders
STRING: Search Tool for the Retrieval of Interacting Genes

## References

[1] Jemal A, Bray F, Center MM (2011). Global cancer statistics. CA Cancer J Clin.

[2] Ferlay J, Steliarova-Foucher E, Lortet-Tieulent J (2013). Cancer incidence and mortality patterns in Europe: estimates for 40 countries in 2012. Eur J Cancer.

[3] Argiris A, Karamouzis MV, Raben D, Ferris RL (2008). Head and neck cancer. Lancet..

[4] Viana LS, Silva FCA, Jacome AAA (2016). Efficacy and safety of a cisplatin and paclitaxel induction regimen followed by chemoradiotherapy for patients with locally advanced head and neck squamous cell carcinoma. Head Neck.

[5] Iyer NG, Tan DS, Tan VK (2015). Randomized trial comparing surgery and adjuvant radiotherapy versus concurrent chemoradiotherapy in patients with advanced, nonmetastatic squamous cell carcinoma of the head and neck: 10-year update and subset analysis. Cancer..

[6] Colombo M, Raposo G, Théry C (2014). Biogenesis, secretion, and intercellular interactions of exosomes and other extracellular vesicles. Annu Rev Cell Dev Biol.

[7] Yáñez-Mó M, Siljander PRM, Andreu Z (2015). Biological properties of extracellular vesicles and their physiological functions. J Extracell Vesicles.

[8] Hong CS, Muller L, Whiteside TL, Boyiadzis M (2014). Plasma exosomes as markers of therapeutic response in patients with acute myeloid leukemia. Front Immunol.

[9] Tkach M, Théry C (2016). Communication by extracellular vesicles: where we are and where we need to go. Cell..

[10] Azmi AS, Bao B, Sarkar FH (2013). Exosomes in cancer development, metastasis, and drug resistance: a comprehensive review. Cancer Metastasis Rev.

[11] Principe S, Hui AB, Bruce J (2013). Tumor-derived exosomes and microvesicles in head and neck cancer: implications for tumor biology and biomarker discovery. Proteomics..

[12] Ramirez MI, Amorim MG, Gadelha C (2018). Technical challenges of working with extracellular vesicles. Nanoscale..

[13] Whiteside TL (2016). Exosomes and tumor-mediated immune suppression. J Clin Invest.

[14] Ludwig S, Floros T, Theodoraki MN (2017). Suppression of lymphocyte functions by plasma exosomes correlates with disease activity in patients with head and neck Cancer. Clin Cancer Res.

[15] Bergmann C, Strauss L, Wang Y (2008). T regulatory type 1 cells in squamous cell carcinoma of the head and neck: mechanisms of suppression and expansion in advanced disease. Clin CancerRes.

[16] Filipazzi P, Burdek M, Villa A, Rivoltini L, Huber V (2012). Recent advances on the role of tumor exosomes in immunosuppression and disease progression. SeminCancerBiol..

[17] Peinado H, Aleckovic M, Lavotshkin S (2012). Melanoma exosomes educate bone marrow progenitor cells toward a pro-metastatic phenotype through MET. NatMed..

[18] Lefebvre JL, Ang KK (2009). Larynx preservation clinical trial design: key issues and recommendations--a consensus panel summary. Head Neck..

[19] Théry C, Ostrowski M, Segura E (2009). Membrane vesicles as conveyors of immune responses. Nat Rev Immunol.

[20] Tan KH, Tan SS, Sze SK (2014). Plasma biomarker discovery in preeclampsia using a novel differential isolation technology for circulating extracellular vesicles. Am J Obstet Gynecol.

[21] Mi H, Huang X, Muruganujan A (2017). PANTHER version 11: expanded annotation data from gene ontology and Reactome pathways, and data analysis tool enhancements. Nucleic Acids Res.

[22] Le QT, Sutphin PD, Raychaudhuri S (2003). Identification of osteopontin as a prognostic plasma marker for head and neck squamous cell carcinomas. Clin Cancer Res.

[23] Gehrmann M, Specht HM, Bayer C (2014). Hsp70--a biomarker for tumor detection and monitoring of outcome of radiation therapy in patients with squamous cell carcinoma of the head and neck. Radiat Oncol.

[24] Singh RD, Haridas N, Patel JB (2010). Matrix metalloproteinases and their inhibitors: correlation with invasion and metastasis in oral cancer. Indian J Clin Biochem.

[25] Lin CW, Tseng SW, Yang SF (2012). Role of lipocalin 2 and its complex with matrix metalloproteinase-9 in oral cancer. Oral Dis.

[26] Su CW, Su BF, Chiang WL (2017). Plasma levels of the tissue inhibitor matrix metalloproteinase-3 as a potential biomarker in oral cancer progression. Int J Med Sci.

[27] Sill M, Schröder C, Shen Y, et al. Protein profiling gastric Cancer and neighboring control tissues using high-content antibody microarrays. Microarrays (Basel). 2016;5(3). 10.3390/microarrays5030019 PMC5040966 27600085

[28] Smith L, Watson MB, O'Kane SL, Drew PJ, Lind MJ, Cawkwell L (2006). The analysis of doxorubicin resistance in human breast cancer cells using antibody microarrays. Mol Cancer Ther.

[29] Sreekumar A, Nyati MK, Varambally S (2001). Profiling of cancer cells using protein microarrays: discovery of novel radiation-regulated proteins. Cancer Res.

[30] Arantes LMRB, De Carvalho AC, Melendez ME, Lopes Carvalho A (2018). Serum, plasma and saliva biomarkers for head and neck cancer. Expert Rev Mol Diagn.

[31] Zupancic K, Blejec A, Herman A (2014). Identification of plasma biomarker candidates in glioblastoma using an antibody-array-based proteomic approach. Radiol Oncol.

[32] Righetti PG, Castagna A, Antonucci F (2005). Proteome analysis in the clinical chemistry laboratory: myth or reality?. Clin Chim Acta.

[33] Diamandis EP (2004). Mass spectrometry as a diagnostic and a cancer biomarker discovery tool: opportunities and potential limitations. Mol Cell Proteomics.

[34] Liu T, Qian WJ, Mottaz HM (2006). Evaluation of multiprotein immunoaffinity subtraction for plasma proteomics and candidate biomarker discovery using mass spectrometry. Mol Cell Proteomics.

[35] Patel K, Wen J, Magliocca K (2014). Heat shock protein 90 (HSP90) is overexpressed in p16-negative oropharyngeal squamous cell carcinoma, and its inhibition in vitro potentiates the effects of chemoradiation. Cancer Chemother Pharmacol.

[36] Dewyer NA, Wolf GT, Light E, Worden F, Urba S, Eisbruch A, Bradford CR, Chepeha DB, Prince ME, Moyer J, Taylor J (2014). Circulating CD4-positive lymphocyte levels as predictor of response to induction chemotherapy in patients with advanced laryngeal cancer. Head Neck..

[37] Liu M, Zhang X, Hu CF, Xu Q, Zhu HX, Xu NZ (2014). MicroRNA-mRNA functional pairs for cisplatin resistance in ovarian cancer cells. Chin J Cancer.

[38] Wan B, Dai L, Wang L (2018). Knockdown of BRCA2 enhances cisplatin and cisplatin-induced autophagy in ovarian cancer cells. Endocr Relat Cancer.

[39] Nicholson RI, Gee JM, Harper ME (2001). EGFR and cancer prognosis. Eur J Cancer.

[40] Zhang Y, Molavi O, Su M, Lai R (2014). The clinical and biological significance of STAT1 in esophageal squamous cell carcinoma. BMC Cancer.

[41] Azimian H, Dayyani M, Toossi MTB, Mahmoudi M (2018). Bax/Bcl-2 expression ratio in prediction of response to breast cancer radiotherapy. Iran J Basic Med Sci.

[42] Deep G, Jain AK, Ramteke A (2014). SNAI1 is critical for the aggressiveness of prostate cancer cells with low E-cadherin. Mol Cancer.

[43] Tang X, Jin L, Cao P (2016). MicroRNA-16 sensitizes breast cancer cells to paclitaxel through suppression of IKBKB expression. Oncotarget..

[44] Hsu HW, Wall NR, Hsueh CT (2014). Combination antiangiogenic therapy and radiation in head and neck cancers. Oral Oncol.

[45] Vassilakopoulou M, Psyrri A, Argiris A (2015). Targeting angiogenesis in head and neck cancer. Oral Oncol.

[46] Bauer JA, Trask DK, Kumar B, Los G, Castro J, Lee JS, Chen J, Wang S, Bradford CR, Carey TE (2005). Reversal of cisplatin resistance with a BH3 mimetic, (−)-gossypol, in head and neck cancer cells: role of wild-type p53 and Bcl-xL. Mol Cancer Ther.

[47] Ji X, Jiang C, Liu Y, Bu D, Xiao S (2011). Fas ligand gene transfer effectively induces apoptosis in head and neck cancer cells. Acta Otolaryngol.

[48] Gadhikar MA, Sciuto MR, Alves MV (2013). Chk1/2 inhibition overcomes the cisplatin resistance of head and neck cancer cells secondary to the loss of functional p53. Mol Cancer Ther.

