# Supplementary figures and images for: A preliminary investigation of circulating extracellular vesicles and biomarker discovery associated with treatment response in head and neck squamous cell carcinoma
Source: BMC Cancer. 2019 Apr 23;19:373. doi: 10.1186/s12885-019-5565-9 (PMC6480898; doi:10.1186/s12885-019-5565-9)

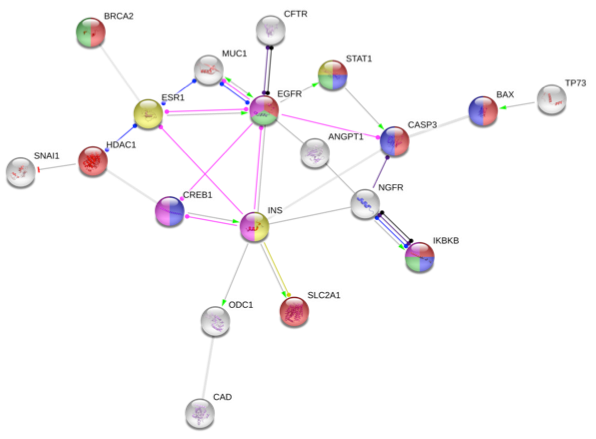

Supplement: Supplementary file 1 — Table S1 List of proteins present in CTB-, AV-EVs and crude plasma of non-responders HNSCC patients. Mean of relative expression was normalized using GenePix Pro 7 software (Molecular Devices). Gene Set Enrichment Analysis (GSEA) algorithm was performed to identify proteins positively related to cancer (+). (TIF 1022 kb) [file 12885_2019_5565_MOESM1_ESM.tif]

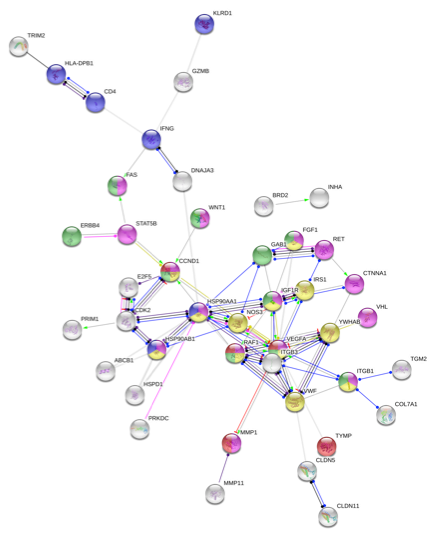

Supplement: Supplementary file 2 — Table S2 List of proteins present in CTB-, AV-EVs and crude plasma of complete responders HNSCC patients. Mean of relative expression was normalized using GenePix Pro 7 software (Molecular Devices). Gene Set Enrichment Analysis (GSEA) algorithm was performed to identify proteins positively related to cancer (+). (TIF 943 kb) [file 12885_2019_5565_MOESM2_ESM.tif]
