# Supplementary material for: A preliminary investigation of circulating extracellular vesicles and biomarker discovery associated with treatment response in head and neck squamous cell carcinoma
Source: BMC Cancer. 2019 Apr 23;19:373. doi: 10.1186/s12885-019-5565-9 (PMC6480898; doi:10.1186/s12885-019-5565-9)
Supplement: Supplementary file 3 — Figure S1 Protein-protein interaction network to specific proteins in EVs from CR patients. Predicted interactions for these proteins (n = 32) were obtained from STRING online database (http://string-db.org). The top five KEGG pathways observed in these proteins were pathways in cancer (red; eight proteins, p = 5.44 × 10− 6), hepatitis B (purple; five proteins, p = 0.0002), pancreatic cancer (green; four proteins, p = 0.0002), prolactin signaling pathway (yellow; four proteins, p = 0.0002) and prostate cancer (lilac; four proteins, p = 0.0004). (DOCX 39 kb) [file 12885_2019_5565_MOESM3_ESM.docx]

**Additional file 1: Table S1.** List of proteins present in CTB-, AV-EVs and crude plasma of non-responders HNSCC patients. Mean of relative expression was normalized using GenePix Pro 7 software (Molecular Devices). Gene Set Enrichment Analysis (GSEA) algorithm was performed to identify proteins positively related to cancer (+).

| **Protein** | **Full Protein Name** | **Swissprot**  **No.** | **Relative Expression** | **Pathway in Cancer - GSEA**  **GSEA** |  |
| --- | --- | --- | --- | --- | --- |
| **CTB-EVs** | | | |  |  |
| BRD2 | Bromodomain-containing protein 2 | P25440 | 7.0 | - | |
| CD68 | Macrosialin | P34810 | 5.5 | - | |
| CD84 | SLAM family member 5 | Q9UIB8 | 13.5 | - | |
| CDK2 | Cyclin-dependent kinase 2 | P24941 | 6.0 | + | |
| CLDN5 | Claudin-5 | O00501 | 6.5 | - | |
| COL7A1 | Collagen alpha-1(VII) chain | Q02388 | 6.0 | - | |
| CR2 | Complement receptor type 2 | P20023 | 5.5 | - | |
| DNAJA3 | DnaJ homolog subfamily A member 3 | Q96EY1 | 7.5 | - | |
| E2F5 | Transcription factor E2F5 | Q15329 | 7.5 | + | |
| ERBB4 | Receptor tyrosine-protein kinase erbB-4 | Q15303 | 6.0 | - | |
| FAS | Tumor necrosis factor receptor superfamily member 6 | P25445 | 9.5 | + | |
| GAB1 | GRB2-associated-binding protein 1 | Q13480 | 5.5 | + | |
| GRM1 | Metabotropic glutamate receptor 1 | Q13255 | 11.0 | - | |
| GSTM1 | Glutathione S-transferase Mu 1 | P09488 | 21.0 | - | |
| GZMB | Granzyme B | P10144 | 6.5 | - | |
| HSPD1 | 60 kDa heat shock protein, mitochondrial | P10809 | 7.5 | - | |
| IRS1 | Insulin receptor substrate 1 | P35568 | 5.5 | - | |
| ITGB1 | Integrin beta-1 | P05556 | 7.0 | + | |
| ITGB3 | Integrin beta-3 | P05106 | 5.5 | + | |
| KLRD1 | Natural killer cells antigen CD94 | Q13241 | 7.0 | - | |
| MKI67 | MKI67 FHA domain-interacting nucleolar phosphoprotein | Q9BYG3 | 6.5 | - | |
| MMP11 | Stromelysin-3 | P24347 | 6.5 | - | |
| MT-CO2 | Cytochrome c oxidase subunit 2 | P00403 | 7.0 | - | |
| MUC5AC | Mucin-5AC | P98088 | 7.0 | - | |
| NEFL | Neurofilament light polypeptide | P07196 | 6.5 | + | |
| NOS3 | Nitric oxide synthase | P29474 | 5.5 | + | |
| PCTK2 | Cyclin-dependent kinase 17 | Q00537 | 7.0 | - | |
| PRKDC | DNA-dependent protein kinase catalytic subunit | P78527 | 8.0 | - | |
| MT-CO2 | Cytochrome c oxidase subunit 2 | P00403 | 6.0 | - | |
| PRTN3 | Myeloblastin | P24158 | 6.5 | - | |
| RAF1 | RAF proto-oncogene serine/threonine-protein kinase | P04049 | 6.5 | + | |
| RET | Proto-oncogene tyrosine-protein kinase receptor | P07949 | 6.5 | + | |
| SIRPA | Tyrosine-protein phosphatase non-receptor type substrate 1 | P78324 | 6.5 | - | |
| SPN | Transcription factor SPN1 | Q06505 | 10 | - | |
| STAT5 | Signal transducer and activator of transcription 5A | P42229 | 6.0 | + | |
| TGM2 | Protein-glutamine gamma-glutamyltransferase 2 | P21980 | 6.0 | - | |
| TNFRSF1B | Tumor necrosis factor receptor superfamily member 1B | P20333 | 8.5 | - | |
| TYMP | Thymidine phosphorylase | P19971 | 21.5 | - | |
| TYR | Tyrosinase | P14679 | 7.0 | - | |
| UBASH3B | Ubiquitin-associated and SH3 domain-containing protein B | Q8TF42 | 6.0 | + | |
| VEGFA | Vascular endothelial growth factor A | P15692 | 7.5 | + | |
| VWF | von Willebrand factor | P04275 | 91.0 | - | |
| WDR48 | WD repeat-containing protein 48 | Q8TAF3 | 8.5 | - | |
| WNT1 | Proto-oncogene Wnt-1 | P04426 | 6.5 | + | |
| YWHAB | 14-3-3 protein beta/alpha | P31946 | 7.5 | - | |
| **AV-EVs** | | | |  |  |
| \| ABCB1 \| \| --- \| | Multidrug resistance protein 1 | P08183 | 34.5 | - | |
| ADRM1 | Proteasomal ubiquitin receptor ADRM1 | Q16186 | 29.0 | + | |
| CASP5 | Caspase-5 | P51878 | 190.5 | - | |
| CCND1 | G1/S-specific cyclin-D1 | P24385 | 33.0 | + | |
| CLDN11 | Claudin-11 | O75508 | 187.0 | - | |
| CTNNA1 | Catenin alpha-1 | P35221 | 35.0 | + | |
| FGF1 | Fibroblast growth factor 1 | P61148 | 70.5 | + | |
| HLA-DP | HLA class II histocompatibility antigen | P04440 | 76.0 | - | |
| HSP90AA1 | Heat shock protein HSP 90-beta | P07900 | 44.0^c^ | + | |
| IGF1R | Insulin-like growth factor 1 receptor | P08069 | 73.0 | + | |
| INHA | Enoyl-[acyl-carrier-protein] reductase [NADH] | P9WGR1 | 53.0 | + | |
| KRT14 | Keratin, type I cytoskeletal 14 | P02533 | 291.0 | + | |
| KRT18 | Keratin, type I cytoskeletal 18 | P05783 | 44.5 | + | |
| SREBF1 | Sterol regulatory element-binding protein 1 | P36956 | 25.5 | - | |
| **Crude Plasma** | | | |  |  |
| ABL1 | Tyrosine-protein kinase ABL1 | P00519 | 68.5 | + | |
| AFP | Alpha-fetoprotein | P02771 | 62.0 | - | |
| AIFM1 | Apoptosis-inducing factor 1, mitochondrial | O95831 | 67.5 | - | |
| ALPL | Alkaline phosphatase, tissue-nonspecific isozyme | P05186 | 39.5 | - | |
| ARF6 | PH and SEC7 domain-containing protein 1 | A5PKW4 | 51.5 | - | |
| BCL2L1 | Bcl-2-like protein 1 | Q07817 | 41.5 | + | |
| CALD1 | Caldesmon | Q05682 | 42.5 | - | |
| CALM1 | Calmodulin-1 | P0DP23 | 42.5 | + | |
| CCNE1 | G1/S-specific cyclin-E1 | P24864 | 55.5 | - | |
| CD24 | Signal transducer CD24 | P24807 | 39.0 | - | |
| CD46 | Membrane cofactor protein | P15529 | 57.0 | - | |
| CDK4 | Cyclin-dependent kinase 4 | P11802 | 47.5 | + | |
| CDK8 | Cyclin-dependent kinase 8 | P49336 | 50.0 | - | |
| CDKN2A | Cyclin-dependent kinase inhibitor 2A | P42771 | 49.5 | + | |
| CFLAR | CASP8 and FADD-like apoptosis regulator | O15519 | 38.5 | - | |
| CLDN1 | Claudin-1 | O95832 | 37.5 | - | |
| CNNE1 | Cyclin E1 | P24864 | 50.5 | + | |
| CUL3 | Cullin-3 | Q13618 | 40.5 | - | |
| CXCR6 | C-X-C chemokine receptor type 6 | O00574 | 39.0 | - | |
| DFFA | DNA fragmentation factor subunit alpha | O00273 | 57.5 | - | |
| DNTT | DNA nucleotidylexotransferase | P04053 | 41.0 | - | |
| DPP4 | Dipeptidyl peptidase 4 | P27487 | 75.0 | - | |
| E2F2 | Transcription factor E2F2 | Q14209 | 86.5 | + | |
| ENO2 | ENO2 | P09104 | 78.5 | - | |
| FCGR2B | Low affinity immunoglobulin gamma Fc region receptor II-b | P31994 | 58.5 | - | |
| FLNA | Filamin-A | P21333 | 92.0 | - | |
| GGT1 | Glutathione hydrolase 1 proenzyme | P19440 | 40.0 | - | |
| GRM5 | Metabotropic glutamate receptor 5 | P41594 | 38.5 | - | |
| HLA-DP |  | P04440 | 79.0 | - | |
| HSPB1 | Heat shock protein beta-1 | P04792 | 58.5 | - | |
| ICOSLG | ICOS ligand | O75144 | 60.0 | - | |
| IL-8 | Interleukin-8 | P10145 | 41.0 | - | |
| IRAK1 | Interleukin-1 receptor-associated kinase 1 | P51617 | 59.0 | - | |
| KRT10 | Keratin, type I cytoskeletal 10 | P13645 | 53.5 | - | |
| KRT13 | Keratin, type I cytoskeletal 13 | P13646 | 53.5 | - | |
| L1CAM | Neural cell adhesion molecule L1 | P32004 | 40.0 | - | |
| LAMB1 | Laminin subunit beta-1 | P07942 | 220.0 | + | |
| LAMB2 | Laminin subunit beta-2 | P55268 | 39.0 | + | |
| LCK | Tyrosine-protein kinase Lck | P06239 | 56.0 | - | |
| MAP2 | Microtubule-associated protein 2 | P11137 | 44.5 | - | |
| MAPK3 | Mitogen-activated protein kinase 3 | P27361 | 49.5 | + | |
| MMP10 | Stromelysin-2 | P09238 | 59.0 | - | |
| MSN | Moesin | P26038 | 49.0 | - | |
| NRG1 | Transcriptional regulator NRG1 | Q5A0E5 | 84.5 | - | |
| PCNA | PCNA-associated factor | PCNA | 72.0 | - | |
| PLCG1 | 1-phosphatidylinositol 4,5-bisphosphate phosphodiesterase gamma-1 | P19174 | 48.5 | + | |
| PTH1R | Parathyroid hormone/parathyroid hormone-related peptide receptor | Q03431 | 41.0 | - | |
| PTPRC | Receptor-type tyrosine-protein phosphatase C | P08575 | 62.0 | - | |
| PXN | Paxillin | P49023 | 75.5 | - | |
| RBL2 | Retinoblastoma-like protein 2 | Q08999 | 58.5 | + | |
| SRF | Serum response factor | A0A024RD16 | 67.5 | - | |
| TFF1 | Trefoil factor 1 | P04155 | 82.5 | - | |
| **CTB-EVs and AV-EVs** | | | |  |  |
| CD4 | T-cell surface glycoprotein CD4 | P01730 | 9.5^a^  1302.5^b^ | + | |
| POLB | DNA polymerase beta | P06746 | 29.0^a^  27.0^b^ | - | |
| PRIM1 | DNA primase small subunit | P49642 | 5.5^a^  337.0^b^ | + | |
| **CTB-EVs and Crude Plasma** | | | |  |  |
| IFNG | Interferon gamma | P01579 | 11.5^a^  46.0^c^ | + | |
| MMP1 | Interstitial collagenase | P03956 | 8.5^a^  37.5^c^ | + | |
| VHL | Protein Vhl | Q9V3C1 | 5.5^a^  38.0^c^ | + | |
| **AV-EVs and Crude Plasma** | | | |  |  |
| HLA-DP | HLA class II histocompatibility antigen | P04440 | 76.0^b^  79.0^c^ | - | |
| HSP90AB1 | Heat shock protein HSP 90-beta | P08238 | 1037.0^b^  39.0^c^ | + | |

Legend: NR (Non-Responders); ^a^CTB-EVs; ^b^AV-EVs; ^c^ Plasma
