# Supplementary material for: A preliminary investigation of circulating extracellular vesicles and biomarker discovery associated with treatment response in head and neck squamous cell carcinoma
Source: BMC Cancer. 2019 Apr 23;19:373. doi: 10.1186/s12885-019-5565-9 (PMC6480898; doi:10.1186/s12885-019-5565-9)
Supplement: Supplementary file 4 — Figure S2 Protein-protein interaction network construction by STRING to specific proteins in EVs from NR patients. Predicted interactions for these proteins (n = 67) were obtained from STRING online database (http://string-db.org). Top five KEGG pathways related to specific proteins in NR-EVs were pathways in cancer (red; 15 proteins, p = 1.74 × 10− 11), PI3K/AKT signaling pathway (purple; 12 proteins, p = 8.4 × 10− 8), proteoglycans in cancer (green; ten proteins, p = 1.58 × 10− 7), antigen processing and presentation (yellow; six proteins, p = 4.56 × 10− 6) and bladder cancer (lilac; five proteins, p = 5.34 × 10− 6). (DOCX 25 kb) [file 12885_2019_5565_MOESM4_ESM.docx]

**Additional file 2: Table S2.** List of proteins present in CTB-, AV-EVs and crude plasma of complete responders HNSCC patients. Mean of relative expression was normalized using GenePix Pro 7 software (Molecular Devices). Gene Set Enrichment Analysis (GSEA) algorithm was performed to identify proteins positively related to cancer (+).

| **Proteins** | | **Full Protein Name** | | | | **Swissprot**  **No.** | | **Relative**  **Expression** | | | | |  | | | | **Pathway in Cancer - GSEA** | | | | | | | |
| --- | --- | --- | --- | --- | --- | --- | --- | --- | --- | --- | --- | --- | --- | --- | --- | --- | --- | --- | --- | --- | --- | --- | --- | --- |
| **CTB-EVs** | | | |  |  | |  | | | |  |  | | | |  | | | | | |  | | |
| ANGPT1 | | Angiopoietin-1 | | | | Q15389 | | 4.0 | | | | | |  | | | | - |  | | | | | |
| BAX | | Apoptosis regulator BAX | | | | Q07812 | | 21 | | | | | |  | | | | + | | | | | |  |
| CAD | | CAD protein | | | | P27708 | | 5.0 | | | | | |  | | | | - | | | | | |  |
| CDH3 | | Cadherin-3 | | | | P22223 | | 5.0 | | | | | |  | | | | - | | | | | |  |
| CASP3 | | Caspase-3 | | | | P42574 | | 21.0 | | | | | |  | | | | + | | | | | |  |
| CREB1 | | Cyclic AMP-responsive element-binding protein 1 | | | | P16220 | | 6.5 | | | | | |  | | | | + | | | | | |  |
| GABRB1 | | Gamma-aminobutyric acid receptor subunit beta-1 | | | | P18505 | | 4.0 | | | | | |  | | | | - | | | | | |  |
| HDAC1 | | Histone deacetylase 1 | | | | Q13547 | | 7.5 | | | | | |  | | | | + | | | | | |  |
| KRT16 | | Keratin, type I cytoskeletal 16 | | | | P08779 | | 4.5 | | | | | |  | | | | - | | | | | |  |
| NGFR | | Tumor necrosis factor receptor superfamily member 16 | | | | P08138 | | 4.5 | | | | | |  | | | | - | | | | | |  |
| PRCC | | Proline-rich protein PRCC | | | | Q92733 | | 13.0 | | | | | |  | | | | - | | | | | |  |
| SLC2A1 | | Solute carrier family 2, facilitated glucose transporter member 1 | | | | P11166 | | 6.0 | | | | | |  | | | | + | | | | | |  |
| TNFSF11 | | Tumor necrosis factor ligand superfamily member 11 | | | | O14788 | | 4.0 | | | | | |  | | | | - |  | | | | |  |
| TP73 | | Tumor protein p73 | | | | O15350 | | 62.5 | | | | | |  | | | | + |  | | | | |  |
| TXNRD2 | | Thioredoxin reductase 2, mitochondrial | | | | Q9NNW7 | | 4.0 | | | | | |  | | | | - |  | | | | |  |
|  | | | |  | |  | | | |  |  | | | |  | | | | | |  | |  |  |
| **AV-EVs** |  | | | |  | |  | | | |  |  | | | |  | | | | | |  | |  |
| BRCA2 | | Breast cancer type 2 susceptibility protein | | | | P51587 | |  | | 6.0 | | | |  | | | | + | |  |  |  |  |  |
| CCNC | | Cyclin-C | | | | P24863 | |  | | 19.0 | | | |  | | | | - | |  |  |  |  |  |
| CFTR | | Cystic fibrosis transmembrane conductance regulator | | | | P13569 | |  | | 11.0 | | | |  | | | | - | |  |  |  |  |  |
| EGFR | | Epidermal growth factor receptor | | | | P00533 | |  | | 16.5 | | | |  | | | | + | |  |  |  |  |  |
| ESR1 | | Estrogen receptor | | | | P03372 | |  | | 6.5 | | | |  | | | | - | |  |  |  |  |  |
| FLG | | Filaggrin | | | | P20930 | |  | | 7.0 | | | |  | | | | - | |  |  |  |  |  |
| GRIA1 | | Glutamate receptor 1 | | | | P42261 | |  | | 15.5 | | | |  | | | | - | |  |  |  |  |  |
| IKBKB | | Inhibitor of nuclear factor kappa-B kinase subunit beta | | | | O14920 | |  | | 11.0 | | | |  | | | | + | |  |  |  |  |  |
| INS | | Insulin | | | | P01308 | |  | | 11.0 | | | |  | | | | + | |  |  |  |  |  |
| MAP3K | | Mitogen-activated protein kinase kinase kinase 1 | | | | Q13233 | |  | | 8.0 | | | |  | | | | - | |  |  |  |  |  |
| MUC1 | | Mucin-1 | | | | P15941 | |  | | 7.5 | | | |  | | | | - | |  |  |  |  |  |
| MVP | | Major vault protein | | | | Q14764 | |  | | 17.5 | | | |  | | | | - | |  |  |  |  |  |
| ODC1 | | Ornithine decarboxylase | | | | P11926 | |  | | 9.0 | | | |  | | | | - | |  |  |  |  |  |
| PAWR | | PRKC apoptosis WT1 regulator protein | | | | Q96IZ0 | |  | | 6.5 | | | |  | | | | - | |  |  |  |  |  |
| STAT1 | | Signal transducer and activator of transcription 1-alpha/beta | | | | P42224 | |  | | 7.5 | | | |  | | | | + | |  |  |  |  |  |
| TPM1 | | Tropomyosin alpha-1 chain | | | | P09493 | |  | | 17.5 | | | |  | | | | - | |  |  |  |  |  |
|  |  | | | |  | |  | | | |  |  | | | |  | | | | | |  | |  |
| **Crude Plasma** | | | | | | |  | | | |  |  | | | |  | | | | | |  | |  |
| BAG1 | | | BAG family molecular chaperone regulator 1 | | | Q99933 | | | 290.0 | | | | |  | | | | + | | |  |  |  |  |
| CHEK1 | | | Serine/threonine-protein kinase Chk1 | | | O14757 | | | 221.5 | | | | |  | | | | + | | |  |  |  |  |
| IVL | | | Involucrin | | | P07476 | | | 193.5 | | | | |  | | | | - | | |  |  |  |  |
| TNFRSF10B | | | Tumor necrosis factor receptor superfamily member 10B | | | O14763 | | | 172.5 | | | | |  | | | | + | | |  |  |  |  |
| TRAP1 | | | Heat shock protein 75 kDa, mitochondrial | | | Q12931 | | | 120.0 | | | | |  | | | | - | | |  |  |  |  |
| VCL | | | Vinculin | | | P18206 | | | 240.0 | | | | |  | | | | - | | |  |  |  |  |
|  | | | |  | | | | | | |  |  |  |  |  |  |  |  |  |  |  |  |  |  |
| **CTB and AV-EVs** | | | | | | |  | | | |  |  | | | |  | | | | | | |  |  |
| SNAI1 | | Zinc finger protein SNAI1 | | | | O95863 | | 5^a,b^ | | | | | | |  | | | - | | | | |  |  |

Legend: CR (Complete Responder); ^a^CTB-EVs; ^b^AV-EVs
